# The Effect of Culture on Human Bone Marrow Mesenchymal Stem Cells: Focus on DNA Methylation Profiles

**DOI:** 10.1155/2016/5656701

**Published:** 2016-01-06

**Authors:** Angela Bentivegna, Gaia Roversi, Gabriele Riva, Laura Paoletta, Serena Redaelli, Mariarosaria Miloso, Giovanni Tredici, Leda Dalprà

**Affiliations:** ^1^School of Medicine and Surgery, University of Milano-Bicocca, 20052 Monza, Italy; ^2^Neurology Unit, Milan Center for Neuroscience (Neuro-MI), University of Milano-Bicocca, 20052 Monza, Italy; ^3^Medical Genetics Laboratory, San Gerardo Hospital, 20052 Monza, Italy

## Abstract

Human bone marrow mesenchymal stem cells (hBM-MSCs) are the best characterized multipotent adult stem cells. Their self-renewal capacity, multilineage differentiation potential, and immunomodulatory properties have indicated that they can be used in many clinical therapies. In a previous work we studied the DNA methylation levels of hBM-MSC genomic DNA in order to delineate a kind of methylation signature specific for early and late passages of culture. In the present work we focused on the modification of the methylation profiles of the X chromosome and imprinted loci, as sites expected to be more stable than whole genome. We propose a model where cultured hBM-MSCs undergo random modifications at the methylation level of most CGIs, nevertheless reflecting the original methylation status. We also pointed out global genome-wide demethylation connected to the long-term culture and senescence. Modification at CGIs promoters of specific genes could be related to the decrease in adipogenic differentiation potential. In conclusion, we showed important changes in CGIs methylation due to long-term* in vitro* culture that may affect the differentiation potential of hBM-MSCs. Therefore it is necessary to optimize the experimental conditions for* in vitro* expansion in order to minimize these epigenetic changes and to standardize safer procedures.

## 1. Introduction

Mesenchymal stem cells (MSCs) are multipotent adult stem cells with self-renewal capacity and the ability to differentiate not only into mesodermal lineages (osteogenic, adipogenic, and chondrogenic lineages), but also towards endodermal or ectodermal derivatives. The multilineage differentiation potential and immunomodulatory properties of MSCs have indicated that they can be used in many clinical therapies, such as tissue engineering, regenerative medicine, autoimmune diseases, and pathologies characterized by chronic inflammatory processes [1, 2].

MSCs from bone marrow (BM-MSCs) are the best characterized adult stem cells but MSC-like populations can be isolated from a variety of different tissues [3]. For MSCs' clinical applications, an adequate number of cells are necessary, and considering the low number of hBM-MSCs in the bone marrow (0.01–0.001%) [4], an* in vitro* expansion phase is required after their isolation. The differentiation capacity of human BM-MSCs (hBM-MSCs) is related to* in vivo* and* in vitro* BM-MSC aging [5]. Loss of MSC osteogenic and adipogenic potential with aging has been demonstrated* in vitro* [6, 7], but no significant differences in osteogenic and adipogenic potentials were detected in aged versus young MSC* in vivo* [7, 8]. Regarding* in vitro* chondrogenic differentiation, the potential of hBM-MSCs was enhanced using cells at passages only between 3 and 6, indicating that this type of mesengenic differentiation is strictly influenced by a limited range of culture passage [9].

However, the need of* in vitro* expansion and/or differentiation of human BM-MSCs (hBM-MSCs) before administration to a patient confers a risk because the high proliferation rate in an artificial cell culture environment could favor the occurrence of genetic and epigenetic alterations. It is generally known that chromosomal aberrations accumulate with age. We and others had argued in favor of a general chromosomal stability of hBM-MSCs, which under prolonged culturing showed progressive growth arrest and entered senescence, without evidence of transformation [10–12]. On the other hand, specific and reproducible epigenetic changes were acquired by hMSCs during* ex vivo* expansion [13]. DNA methylation (DNAm) patterns were overlapping and maintained throughout both long-term culture and aging, and highly significant differences were observed only at specific CpG islands (CGIs), associated with promoter regions, especially in homeobox genes and genes involved in cell differentiation [14].

In this work we focused our attention on DNA methylation profiles of chromosomes of cultured hBM-MSCs in order to compare their state in early and late passages. In particular, we evaluated sites in the genome that are generally considered to be more evolutionarily complex and epigenetically stable loci (imprinted and X chromosome genes) where only a single allele is normally methylated, compared to most genes where the pattern of DNA methylation is identical on both alleles [15].

## 2. Methods

### 2.1. MeDIP-Chip

Data processing in this work started from the MeDIP-CGI-array experiments carried out on hBM-MSCs obtained from healthy donors after the* acquisition of a written informed consent* (original dataset available on request) [10]. Methylated DNA immunoprecipitation and chip hybridization were performed following the guidelines of Agilent Microarray Analysis of Methylated DNA Immunoprecipitation Protocol (Version 1.0, Agilent Technologies, Santa Clara, CA, USA). Methylation analysis was performed on a genomic equimolar pool of DNA of hBM-MSCs from four different donors: donor 1 and donor 2 at P3; donor 5 and donor 6 at P6 (pool of early passages); donor 1 and donor 6 at P9; donor 2 at P10; donor 5 at P12 (pool of late passages). The two pools were used in two independent experiments as reference samples. Briefly, purified genomic DNA was sonicated to fragments of 200–600 bp in size and 5 *μ*g of sheared DNA was immunoprecipitated using 50 *μ*L of pan-mouse IgG Dynal magnetic beads (Life Technologies Italia, Monza, Italy) and 5 *μ*g of 5-methylcytosine antibody (Eurogentec, Seraing, Belgium). DNA was eluted and then purified by phenol : chloroform procedure and precipitated with ethanol. Neither MeDIPed DNA nor reference DNA was amplified but they were directly labeled with Cyanine 5- and Cyanine 3-dUTP nucleotides, respectively, using Agilent Genomic DNA Labeling Kit Plus (Agilent Technologies, Santa Clara, CA, USA). Labeled DNA was cleaned up using MicroconTM YM-30 columns (Millipore, Billerica, MA, USA) and eluted in Tris-EDTA (TE) buffer. Cy5- and Cy3-labeled samples were combined in a single mixture and hybridized onto a human CGI-array 1 × 244 K (Agilent Technologies) for 40 hs at 67°C. The array contains 237,220 probes (45 to 60 mer) representative of all 27,639 CGIs in the human genome, at a density of about 1 probe per 100 bp. The* pseudoautosomal regions* (PAR1 and PAR2) of the human X chromosome are not included.

Microarrays were scanned using an Agilent microarray scanner and images analyzed with Agilent Feature Extraction software v10.7. Raw data, expressed as combined *z*-score (*P* value), were assigned by Agilent Genomic Workbench 6.5 and further analyzed according to the methodological approach conceived by Straussman et al. [16]. For each experiment, a bimodal methylation curve was derived: the probe *Z*-scores for each island were averaged to obtain the Island Methylation Score (IMS) on the *x* axis, whereas the number of probes was on the *y* axis. We then set numeric thresholds for determining the methylation status of each island. We calculated the distance between the demethylated (H1) and methylated (H2) peaks and set the upper and lower limits for DNA methylation as ±10% of this value from the IMS at the lowest point (L), located between the two peaks in the bimodal distribution curve. Islands with an IMS above the upper threshold were assigned a value of +1 (methylated), whereas islands with an IMS below the lower threshold were assigned a value of −1 (demethylated). Islands with an IMS between the two thresholds were considered undetermined (0) and were excluded from subsequent analyses.

#### 2.1.1. Selection of CGIs Associated with X-Inactivated Genes

We extrapolated a list of 199 genes, from a total of 293 probes methylated in a manner consistent with X chromosome inactivation (XCI), from Table S5 (sheet C) published by Nazor et al. [15], who identified X chromosome CpG sites subjected to XCI by a 27K DNA methylation array. 

*Selection of CGIs Associated with Genes Escaping XCI*. We extrapolated a list of 52 high confidence genes predicted to escape XCI based on differential methylation of human active and inactive X chromosomes from Supplementary Table  3 published by Sharp et al. [17] (column high confidence predictions: female methylation < 0.65 and methylation difference < 0.39). 

*DNA Methylation of Imprinted Genes*. We extrapolated a list of 37 genes listed in http://www.geneimprint.com/site/genes-by-species and included in our MeDIP-CGI-array experiments [10]. The function of each gene was verified by consulting UCSC http://genome.ucsc.edu/. We used the GOstat software http://gostat.wehi.edu.au/ [18] to attribute a specific GO term. GO analysis was selected for the biologic processes, and a *P* < 0.05 was imposed. 

*DNA Methylation of Stemness and Differentiation-Related Genes*. We considered the gene list of the Human Mesenchymal Stem Cell RT² Profiler PCR Array, which profiles the expression of 84 key genes involved in maintaining pluripotency and self-renewal status. These 84 genes were grouped in four functional classes: (1) Stemness Markers; (2) MSC-Specific Markers; (3) Other Genes Associated with MSC; (4) MSC Differentiation Markers. For the complete gene list see http://www.qiagen.com/products/catalog/assay-technologies/real-time-pcr-and-rt-pcr-reagents/rt2-profiler-pcr-arrays?catno=PAHS-082Z#geneglobe. 18 of 84 genes were eliminated from this list because they were not present in our MeDIP-CGI-array experiments [10].

All the statistical analyses were performed by using a chi-square test.

## 3. Results

### 3.1. The X Chromosome Is Not More Stable than the Other Chromosomes in Culture

We previously showed that, during* in vitro* culture, differently from most other chromosomes, the X chromosome does not change the status of global methylation, maintaining a prevalent methylated profile [10]. Indeed, we computed that, on a total of 665 CGIs, the ratio between methylated and unmethylated CGIs is similar in early and late passages (1.7 versus 1.6, resp.), while the same ratio computed on the whole autosomes shifted from 1.59 in early to 0.82 in late passages, attesting that a global demethylation affects CGIs during the culture. However, by a deeper analysis we noted that 33.2% (221) out of the X chromosome CGIs changed the methylation status during* in vitro* culture, reversing from a methylated to a demethylated status (116 CGIs newly demethylated) or vice versa (105 CGIs newly methylated). This percentage does not differ from that of autosomes (35.6%) or chromosome 20 that has a similar number of CGIs to the X chromosome (737 CGIs) and shows a reversal of the methylation status in 31.4% of the total CGIs (Table 1).

**Figure 1:**
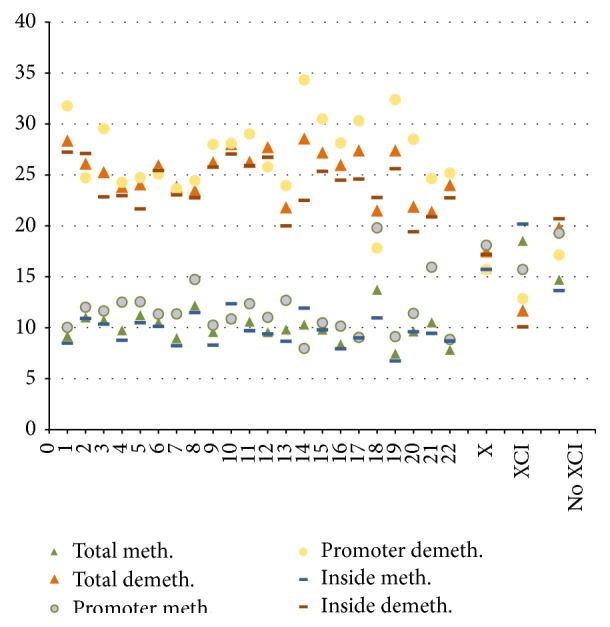
Percentage of total, promoter, and inside CGIs reversing the methylation status. The percentage of CGIs reversing the methylation status (*y*-axis) is indicated for each chromosome (*x* axis). Cold colors identify newly methylated CGIs; warm colors identify newly demethylated CGIs. XCI: CGIs associated with genes subjected to X inactivation. No XCI: CGIs not associated with XCI genes.

But if we consider CGIs that have reversed the methylation status during* in vitro* culture, the behavior of the X chromosome significantly differs from the autosomes (see Table 1 and Figure 1) for the following reasons.

(i) For the autosomes prevail changes towards an unmethylated profile (Figure 1) as shown also by the low ratio between newly methylated and newly demethylated CpGi (0.37) in Table 1, while this ratio for the X chromosome is significantly shifted close to 1 (0.9), due to the comparable percentage of CGIs which reversed to methylated and to unmethylated status (15.7% and 17.4%, resp.); indeed in comparison to the autosomes, not only did a significant lower percentage of X chromosome CGIs become demethylated (25.8% in all the autosomes versus 17.4% in the X chromosome) but also higher percentage became methylated from an unmethylated status (9.7% in all the autosomes versus 15.7% in the X chromosome) (*P* < 0.01 for both hypotheses), in both promoter and inside CGIs. A similar profile is observed among all the autosomes with the exception of chromosome 18 (Figure 1), characterized by a very low percentage of new promoter CGIs demethylation and a very high percentage of* de novo* promoter CGI methylation.

(ii) Unlike most autosomes, where promoter CGIs are significantly more modulated than inside CGIs (38.8% versus 33.8%, considering all the autosomes), the percentage of reversal methylation does not change significantly for the X chromosome considering individually the two classes of CGIs (33.8% versus 32.9%).

### 3.2. Genes Subjected to X Chromosome Inactivation and Genes Predicted to Escape It Are Not More Stable than Other Genes but Show Peculiar Profiles

We checked whenever genes subjected to X chromosome inactivation (XCI) or escaping XCI show a more stable profile than all other X linked genes.

We selected from our data [10] 152 genes included in a list of 199 genes known to be subjected to XCI [15]. 189 CGIs (within 665 belonging to the X chromosome) were associated with these 152 selected genes. In this category, 30.1% out of 189 CGIs showed reversal methylation, with a ratio of 1.59 between newly methylated and newly demethylated; similarly, 34.4% out of the remaining 476 CGIs (not associated with XCI genes) reversed methylation, but with a ratio of 0.74 between newly methylated and newly demethylated (Table 1 and Figure 1). These data show that in CGIs associated with XCI genes a* de novo in vitro* methylation prevails on* de novo* demethylation and that this pattern is especially evident at inside CGIs of XCI genes in comparison with inside CGIs of other X linked genes.

**Figure 2:**
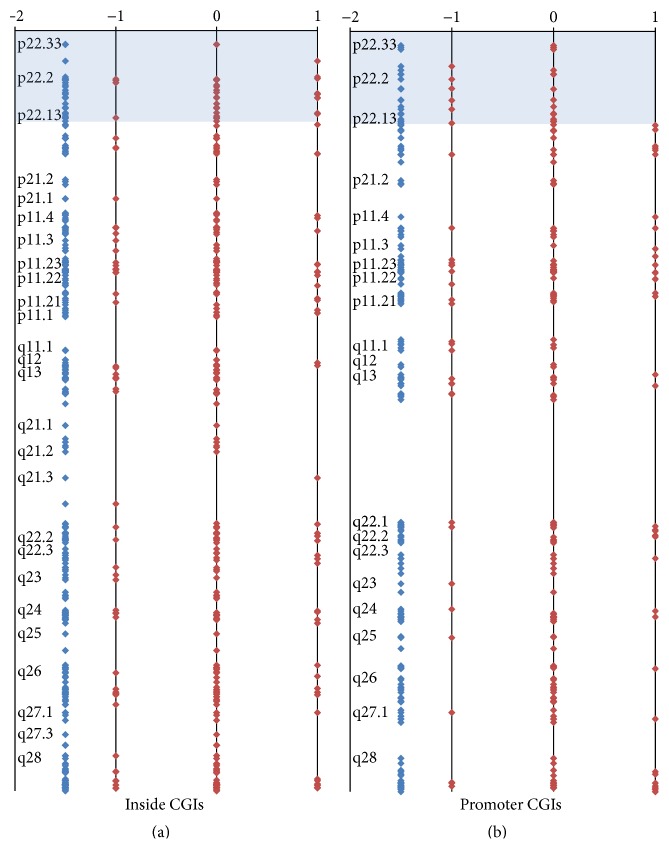
X chromosome CGIs profiles (promoter versus inside). (a) CGIs associated with gene inside; (b) CGIs associated with gene promoter. Each dot corresponds to a CGI in the X chromosome map; blue dots: CGIs represented in the array; red dots: CGI status after* in vitro* culture. *x* axis: dots located at 0 correspond to islands that did not change the methylation status after culture; dots at 1: CGIs that reversed the methylation status from unmethylated to methylated after culture; dots at −1: CGIs that reversed the methylation status from methylated to unmethylated after culture. Light blue area defines a region of 18 Mb at Xp22.33-22.13 characterized by absence of* de novo* methylation in promoter CGIs.

The same approach was applied for genes escaping XCI: we matched a list of 52 genes predicted to escape XCI [17] to our data, thus selecting a group of 24 genes associated with 26 CGIs. We found that 38.4% out of these 26 CGIs reversed the methylation status (Table 1). No data on the percentage of reversal methylation to newly methylated/demethylated CGIs was calculated, due to the small sample. As genes escaping XCI represent only about the 15% of all X linked genes and they are clustered [19], we analyzed a plot profile of the X chromosome (Figure 2), comparing inside versus promoter CGIs. It appears evident that promoter CGIs mapping at Xp22.33-22.13 (hg18:2758138-18913158) are not subjected to* de novo* methylation during* in vitro* culture, differently from inside CGIs. This genomic interval, which does not include the* pseudoautosomal region* (PAR) which is not represented in our MeDIP-CGI-array, corresponds to the major clustering of escaping XCI genes [19].

### 3.3. DNA Methylation of Imprinted Genes: Early versus Late Passages in Culture

By matching a list of 95 known imprinted genes to our data, we identified 37 imprinted genes, of which 23 (62.1%) remained unvaried after* in vitro* culture, while 14 (37.8%) reversed their methylation status (Supplemental Table  1) (see in the Supplementary Material available online at http://dx.doi.org/10.1155/2016/5656701). 17 of the 37 genes express the paternal allele and 14 the maternal one. For 4 genes the methylation status depends on the isoform and for 2 of them it remains unknown.

Among the genes that express the paternal allele, 12 remained unchanged (70.5%), 4 lost their methyl groups (23.5%), and only one (5.8%) acquired a methylated status. Maternally expressed imprinted genes, instead, changed their methylation status in 9/14 cases (64%, 7 reversing towards methylation and 2 towards unmethylation), while only 5 of them (36%) remained unchanged.

**Figure 3:**
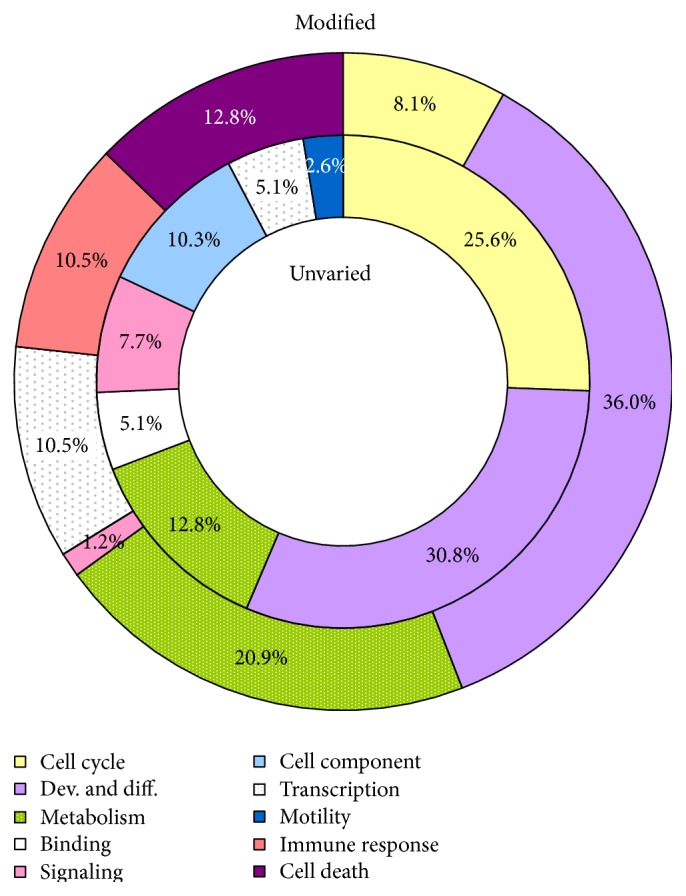
Percentage representativeness of biological processes: unvaried (inner ring) versus modified imprinted genes (outer ring). The genes with an unvaried methylation status (inner circle) and the modified ones (outer circle) were analyzed by GOstat software to determine their functional involvement in cell biology. The classes, in which a variable number of GO terms were pooled, were arbitrarily determined and represent the main biological processes taking place within a cell, as reported in the legend box.

Moreover, we applied the GOstat* software* to both unvaried and modified imprinted genes to classify the genes into specific GO terms which have been further grouped into more general biological processes (Figure 3). Comparing the percentages of biological processes involving unvaried versus modified genes, some peculiar aspects emerge: (i) a general shift of the percentages for common functional categories (i.e., cell cycle, transcription, metabolism, and signaling); (ii) the disappearance of some biological classes, such as binding, cell component, and motility; and (iii) the emergence of new categories (immune response and cell death). It is also noteworthy that a much lower percentage of unvaried genes are involved in a metabolic process if compared to the same value in modified genes (17.4% versus 71.4%, Tables 2 and 3).

### 3.4. DNA Methylation of Stemness and Differentiation-Related Genes: Early versus Late Passages in Culture

As serial passages of hBM-MSCs in culture may affect their ability to differentiate and proliferate [4–6, 9, 20], we focused our attention on DNA methylation of a list of 65 genes related to stemness and differentiation (see Methods and Tables 4 and 5). Of the 42 genes related to the first category (Stemness Markers, MSC-Specific Markers, and Other Genes Associated with MSC), a total of 49 CGIs were considered, 63% of which were inside. 29 CGIs out of 49 (59%) were unchanged, considering early versus late passages (see Table 6). In these 29 unvaried CGIs 10 (34.5%) were localized into promoters and 18 (62%) were inside. The general trend for the 20 CGIs of 49 that change their DNA methylation status is towards demethylation: 16 undergo a wave of demethylation and only 4 undergo a wave of methylation. No statistically significant differences were observed distinguishing between promoters and inside CGIs.

The list of genes related to the second category (*differentiation*) includes two genes of the* stemness* class (*BMP2* and* KRD*); thereafter the total is 25. A total of 35 CGIs were considered, 57% of which were located inside. 21 (60%) of the total CGIs were unchanged considering early versus late passages (see Table 7). Also, for this second category of genes, the general trend of the 40% of CGIs that change the DNA methylation status is towards demethylation (12 out of 14 CGIs).

## 4. Discussion

The last decade has witnessed a rapidly growing interest in MSC therapy reflected also by the increasing number of clinical studies associated with these multipotent stromal cells (https://clinicaltrials.gov/). However, MSC-based therapies require an* in vitro* expansion phase after their isolation and considering the low number of hBM-MSCs in the bone marrow a long-term* in vitro* cultivation is needed [4]. The effects of extended* in vitro* cultivation on physiological functions are still poorly understood, although the risk of senescence is well established and is associated with specific epigenetic changes [21, 22]. Moreover, several studies have reported the reduction of differentiation potential in long-term* in vitro* cultured hBM-MSCs [6, 20].

In a previous study we analyzed the DNA methylation levels of a pool of hBM-MSC genomic DNA from four different donors in order to delineate a kind of methylation signature specific for early and late passages, avoiding interindividual differences among donors [10]. We revealed a significant decrease in CGIs methylation levels and a reversal of CGIs methylated and unmethylated percentages, between early and late passages, for almost all chromosomes. As the X chromosome was one of the few maintaining a high constant ratio between total methylated and unmethylated CGIs, we thought it may be due to a more stable propagation in mitosis of the X chromosome methylation patterns.

To deepen this aspect, in this work we focused on modification of the methylation profile of the X chromosome and imprinted loci, as sites expected to be more stable than whole genome, in order to evaluate the effects of long-term* in vitro* culture on DNA methylation stability.

Our data show that, after* in vitro* culture, X linked and imprinted genes are not more stable than other autosomal genes, all showing a similar and high percentage of CGIs which reverse the methylation status. We demonstrated that the DNA methylation stability of the X chromosome was merely apparent and is due to a similar amount of newly methylated and newly demethylated CGIs. Conversely in the autosomes high amount of newly demethylated CGIs was responsible for the switch to an overall unmethylated profile. In comparison to the autosomes, the X chromosome not only showed a significant lower percentage of newly demethylated CGIs, but also a significant higher percentage of newly methylated CGIs.

If* in vitro* culture affects the genome as a global modifying force, chromosomes with similar starting level of methylation will not be substantially different in late passages. Instead, X chromosome CGIs seem to resist demethylation and be prone to methylation. We know that the ratio of the number of methylated and unmethylated CGIs in early passages is similar between the X chromosome and all the autosomes (1.72 versus 1.59), but we do not know the level of methylation, as MeDIP-CGI-array approach can only assess a qualitative (methylated or unmethylated) but not a quantitative measure of the methylation status for each CGI. To explain the peculiar behavior of the X chromosome we speculate that at early passages the X chromosome CGIs are quantitatively more methylated than the autosomal ones. In this view, these hypermethylated X chromosome CGIs would be more resistant to* in vitro* demethylation wave than the autosomal ones (i.e., a lower X chromosome CGIs percentage reversed from the methylated to the unmethylated status). At the same manner, unmethylated X chromosome CGIs have a relative higher methylation level compared to the autosomal ones, making them more prone to shift towards a methylated profile (i.e., a higher X chromosome CGIs percentage reversed from the unmethylated to the methylated status). Likewise XCI genes, which are expected to be relatively more methylated than other X linked genes, show an even higher ratio between newly methylated/demethylated CGIs. Accordingly, we expect that CGIs of genes escaping XCI, known to have methylation levels indistinguishable from autosomal genes [23], have a tendency towards demethylation and a lower* de novo* methylation: that is what we observed for promoter CGIs mapping at Xp22.33-22.13, where we find clustered genes escaping XCI [19].

It is important to note that though MeDIP-CGI-array data were generated from a pool of two XY male combined with two XX female samples, the analyses were conducted by comparing the same pool at early and late passages; therefore the ratio 2 : 1 between active and inactive X chromosomes remains unchanged. The MeDIP-CGI-array approach does not allow allelic discrimination between active and inactive X chromosome loci but detects a mean methylation value between them. That means that we do not know if changes we detected occur in the active, in the inactive, or in both XCI loci; anyway the mean value is expected to be constant during* in vitro* culture.

Chromosome 18 also showed a peculiar profile, with a very high percentage of* de novo* promoter CGI methylation as regards other autosomes. To our knowledge, no data that could explain such a peculiar methylation profile of chromosome 18 corresponding to its behavior* in vitro* are reported so far. However, Zhang et al. [24] reported that a disproportionately high number of clustered upregulated senescence-specific genes were located, among others, on chromosome 18. One hypothesis is that these clustered genes are regulated by methylation because of being involved in senescence of hBM-MSC. GO analysis failed to detect overrepresented GO terms related to senescence within the list of 19 genes of chromosome 18 with a* de novo* promoter CGI methylation (*THOC1*,* YES1*,* EPB41L3*,* NDUFV2*,* AL359580*,* ESCO1*,* RBBP8*,* B4GALT6*,* C18orf34*,* ZNF24*,* C18orf37*,* P15RS*,* BRUNOL4*,* KIAA1632*,* BC041860*,* KIAA0427*,* CCDC11*,* RTTN*, and* BC017478*). However, by consulting the* GenomeRNAi human phenotypes* at the database genecards (http://www.genecards.org/) some of these genes would be related to senescence; for example,* RTTN*,* RBBP8,* and* THOC1* increased gamma-H2AX phosphorylation, while* P15RS* and* YES1* decreased telomerase activity, both markers of aging [25, 26]. Furthermore,* EPB41L3* has been reported to be downregulated in senescent human dermal fibroblasts [27].

As regards stemness and differentiation-related genes, they undergo a decrease in DNA methylation upon long-term culture such as the whole genome. How this is related to senescence induced by* in vitro* culture is not yet understood. However the promoter of* PPARG* gene undergoes a* de novo* methylation upon long-term culture and could be associated with the decrease in adipogenic potential described also in literature [14].

In conclusion, we suggest a model in which cultured hBM-MSCs undergo random modifications of the methylation level of most CGIs, reflecting the status of the methylation in origin. No genomic regions or loci expected to be stable are spared (e.g., the X chromosome and imprinted genes). However, due to limitations of our analytical technique that does not allow the quantification of methylation level, at the moment we cannot prove our hypothesis.

Moreover, we confirmed that a global genome-wide demethylation affects cultured hBM-MSCs, hypothesizing that this phenomenon could be related to senescence of cells. Modification at CGIs promoters of specific genes, such as* PPARG*, could be related to the decrease in adipogenic differentiation potential. Ultimately, optimization of methods to minimize the degree of this epigenetic instability is required. As yet, it is not clear whether instability is due to the supraphysiological levels of methyl group substrates present in culture medium, to other variable media components, to specific passage methods, or to other unknown factors; certainly the risks increase with increasing passages in culture.

## Supplementary Material

DNA Methylation of Imprinted Genes: We extrapolated a list of 37 genes listed in and included in our MeDIP-CGI-array experiments. The function of each gene was verified by consulting UCSC and listed in Supplemental Table 1.

## Figures and Tables

**Table 1:** Percentage and ratio of CGIs reversing the methylation status in culture.

		Total CGIs		Promoter CGIs		Inside CGIs	
	Number of CGIs	% reversal	Ratio met/dem.	% reversal	Ratio met/dem.	% reversal	Ratio met/dem.
X chromosome	665	33.20	0.9^*∗*a^	33.80	1.15	32.90	0.91
All autosomes	23133	35.60	0.37^*∗*a^	38.8^*∗*c^	0.40	33.8^*∗*c^	0.37
Chromosome 20	737	31.40	0.44^*∗*a^	39^*∗*d^	0.40	29^*∗*d^	0.49
XCI genes	189	30.10	1.59^*∗*b^	28.50	1.20	30.20	2^*∗*e^
No XCI genes	476	34.40	0.74^*∗*b^	36.60	1.12	34.30	0.65^*∗*e^
Escaping genes	26	38.40					

^*∗*^ *p* < 0.01 for comparison of value in cells of the same superscripted letters.

**Table 2:** Percentage distribution of unvaried genes.

Cell cycle	*4 genes*	**17.4**%
	cdkn1c; rb1; ndn; dras3	

Development/ differentiation	*10 genes*	**43.5**%
	mest; dgcr6; ndn; tfpi2; ube3a; rb1; kcnq1; cdkn1c; dlgap2; zim2	

Metabolic process	*4 genes*	**17.4**%
	cdkn1c; rb1; diras3; ddc	

Binding	*1 gene*	**4.3**%
	gnas	

Signaling	*3 genes*	**13.0**%
	cdkn1c; ndn; grb10	

Cell component	*11 genes*	**47.8**%
	lin28b; dgcr6; ndn; klf14; fam50b; ube3a; rb1; zim2; cdkn1c; dgcr6l; dlgap2	

Transcription	*3 genes*	**13.0**%
	cdkn1c; rb1; diras3	

Cell motility	*1 gene*	**4.3**%
	ndn	

**Table 3:** Percentage distribution of modified genes.

Cell cycle	*2 genes*	**14.3**%
	tp73; wt1	
Development/ differentiation	*8 genes*	**57.1**%
	atp10a; tp73; ppp1r9a; nlrp2; dlk1; phlda2; dlx5; slc22a2	

Metabolic process	*10 genes*	**71.4**%
	slc22a2; wt1; nlrp2; tp73; atp10a; znf597; dlx5; phlda2; tceb3b; snrpn	

Signaling	*1 gene*	**7.1**%
	tp73	

Immune response	*1 gene*	**7.1**%
	nlrp2	

Cell death	*3 genes*	**21.4**%
	tp73; nlrp2; phlda2	

**Table 4:** DNA methylation status of stemness-related genes in hBM-MSCs: pool of early passages versus pool of late passages.

Category	Genes	Cytoband	Gene region	Methylation status	
				Early	Late
Stemness Markers	FGF2 (bFGF)	4q26	Inside	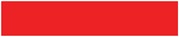	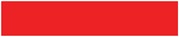
	LIF	22q12.2	Inside	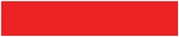	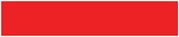
	SOX2	3q26.3	Inside	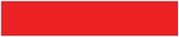	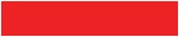
			Downstream	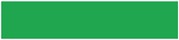	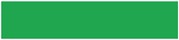
	TERT	5p15.33	Promoter	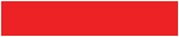	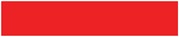
			Inside	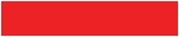	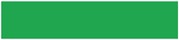
	WNT3A	1q42	Inside	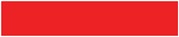	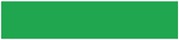
	ZFP42	4q35.2	Promoter	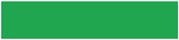	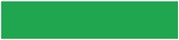

MSC-Specific Markers	ALCAM	3q13.1	Inside	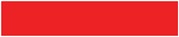	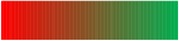
	ANPEP	15q25	Promoter	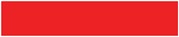	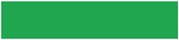
	BMP2	20p12	Inside	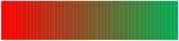	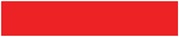
	CASP3	4q34	Inside	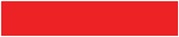	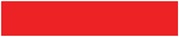
	CD44	11p13	Inside	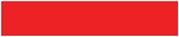	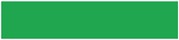
	ENG	9q34.11	Inside	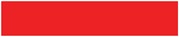	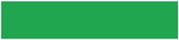
	ERBB2 (HER2)	17q12	Inside	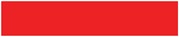	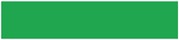
	FUT4	11q21	Inside	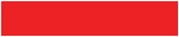	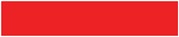
	FZD9	7q11.23	Promoter	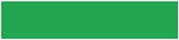	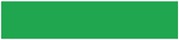
			Inside	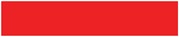	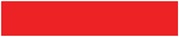
	ITGA6	2q31.1	Inside	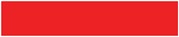	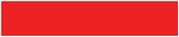
	ITGAV	2q31-q32	Inside	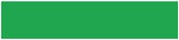	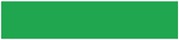
	KDR	4q11-q12	Promoter	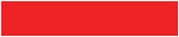	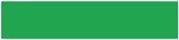
	MCAM	11q23.3	Promoter	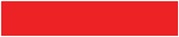	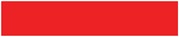
			Inside	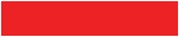	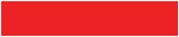
	NGFR	17q21-q22	Inside	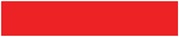	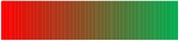
	NT5E	6q14-q21	Inside	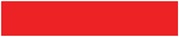	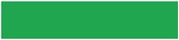
	PDGFRB	5q33.1	Inside	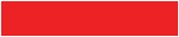	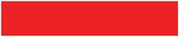
	PROM1	4p15.32	Promoter	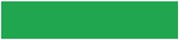	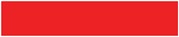
	THY1	11q23.3	Promoter	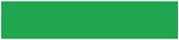	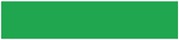
			Inside	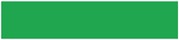	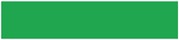

Other Genes Associated with MSCs	ANXA5	4q27	Promoter	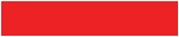	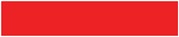
	BDNF	11p13	Promoter	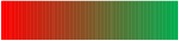	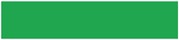
			Inside	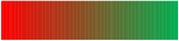	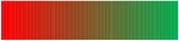
	BMP7	20q13	Promoter	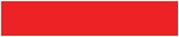	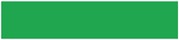
			Inside	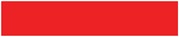	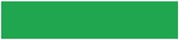
	COL1A1	17q21.33	Promoter	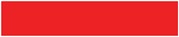	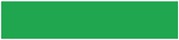
	CTNNB1	3p21	Inside	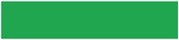	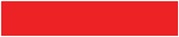
	FUT1	19q13.3	Inside	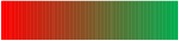	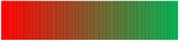
	GTF3A	13q12.3-q13.1	Inside	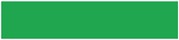	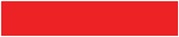
	ICAM1	19p13.3-p13.2	Inside	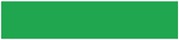	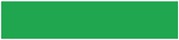
	ITGB1	10p11.2	Promoter	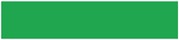	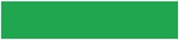
	KITLG	12q22	Promoter	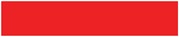	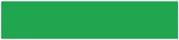
	MMP2	16q13-q21	Inside	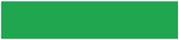	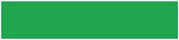
	NES	1q23.1	Promoter	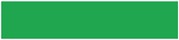	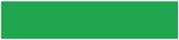
	NUDT6	4q26	Inside	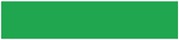	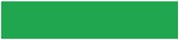
	PIGS	17p13.2	Promoter	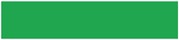	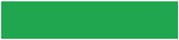
	SLC17A5	6q13	Promoter	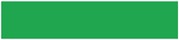	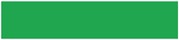
	VEGFA	6p12	Inside	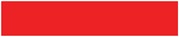	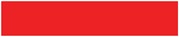
	VIM	10p13	Inside	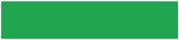	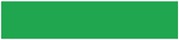
	VWF	12p13.3	Inside	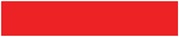	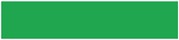

Red: the DNA methylated status prevails over the unmethylated one; green: the DNA unmethylated status prevails over the methylated one; red/green: balance between the two states.

**Table 5:** DNA methylation of differentiation-related genes in hBM-MSCs: pool of early passages versus pool of late passages.

Category	Genes	Cytoband	Gene region	Methylation status	
				Early	Late
MSC Differentiation Markers, osteogenesis	BMP2	20p12	Inside	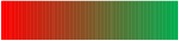	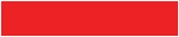
	BMP6	6p24-p23	Inside	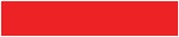	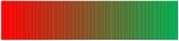
	HDAC1	1p34	Inside	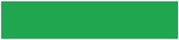	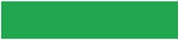
	HNF1A	12q24.2	Inside	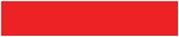	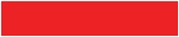
	KDR	4q11-q12	Promoter	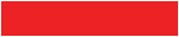	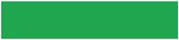
	PTK2	8q24.3	Promoter	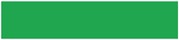	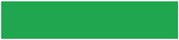
	RUNX2	6p21	Promoter	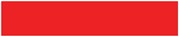	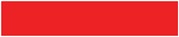
			Inside	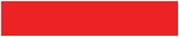	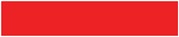
	SMURF1	7q22.1	Promoter	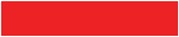	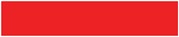
			Inside	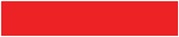	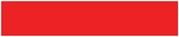
	SMURF2	17q22-q23	Promoter	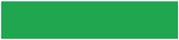	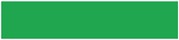
	TBX5	12q24.1	Promoter	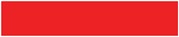	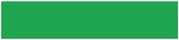
			Inside	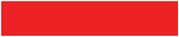	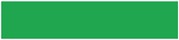

Adipogenesis	PPARG	3p25	Promoter	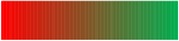	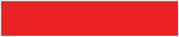
	RUNX2	6p21	Promoter	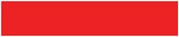	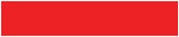
			Inside	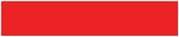	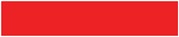

Chondrogenesis	ABCB1 (MDR1)	7q21.12	Inside	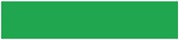	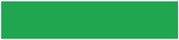
	BMP2	20p12	Inside	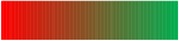	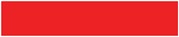
	BMP4	14q22-q23	Promoter	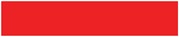	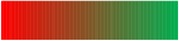
			Inside	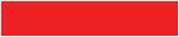	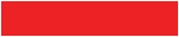
			Downstream	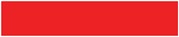	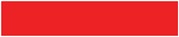
	BMP6	6p24-p23	Inside	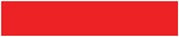	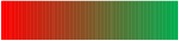
	GDF5 (CDMP-1)	20q11.2	Inside	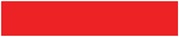	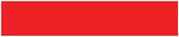
	GDF6	8q22.1	Promoter	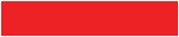	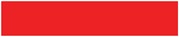
			Inside	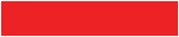	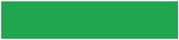
	GDF7	2p24.1	Inside	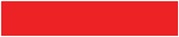	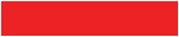
			Downstream	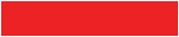	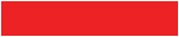
	HAT1	2q31.2-q33.1	Inside	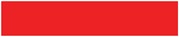	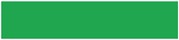
	ITGAX	16p11.2	Inside	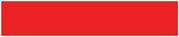	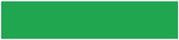
	KAT2B (PCAF)	3p24	Inside	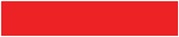	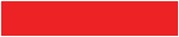
	SOX9	17q23	Promoter	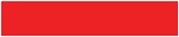	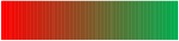
			Inside	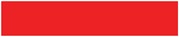	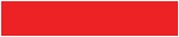
	TGFB1	19q13.1	Promoter	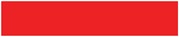	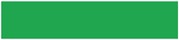
			Inside	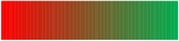	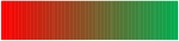

Myogenesis	JAG1	20p12.1-p11.23	Promoter	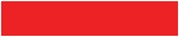	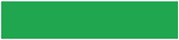
	NOTCH1	9q34.3	Promoter	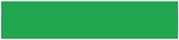	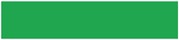
			Inside	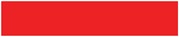	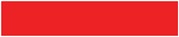

Tenogenesis	BMP2	20p12	Inside	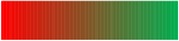	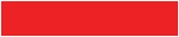
	GDF15 (PLAB)	19p13.11	Inside	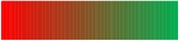	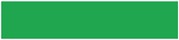
	SMAD4	18q21.1	Inside	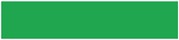	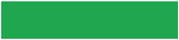
	TGFB1	19q13.1	Promoter	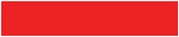	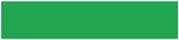
			Inside	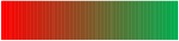	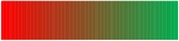

Red: the DNA methylated status prevails over the unmethylated one; green: the DNA unmethylated status prevails over the methylated one; red/green: balance between the two states.

**Table 6:** MSC stemness genes.

Total genes	Total CGIs	Promoter CGIs	Inside CGIs	Downstream CGIs
42	49	17	31	1
Unvaried	29	10	18	1
Unmet wave	16	6	10	0
Met wave	4	1	3	0

**Table 7:** MSC differentiation genes.

Total genes	Total CGIs	Promoter CGIs	Inside CGIs	Downstream CGIs
25	35	13	20	2
Unvaried	21	6	13	2
Unmet wave	12	6	6	0
Met wave	2	1	1	0
