# Supplementary material for: The Effect of Culture on Human Bone Marrow Mesenchymal Stem Cells: Focus on DNA Methylation Profiles
Source: Stem Cells Int. 2016 Jan 6;2016:5656701. doi: 10.1155/2016/5656701 (PMC4736560; doi:10.1155/2016/5656701)
Supplement: Supplementary file 1 — DNA Methylation of Imprinted Genes: We extrapolated a list of 37 genes listed in and included in our MeDIP-CGI-array experiments. The function of each gene was verified by consulting UCSC and listed in Supplemental Table 1. [file 5656701.f1.docx]

***DNA Methylation of Imprinted Genes*.**

We extrapolated a list of 37 genes listed in <http://www.geneimprint.com/site/genes-by-species>

and included in our MeDIP-CGI-array experiments. The function of each gene was verified by consulting UCSC <http://genome.ucsc.edu/> and listed in the table below.

| **Supplemental Table 1. Methylation status of imprinted genes at early vs late passages in culture.** | | | | | | | |
| --- | --- | --- | --- | --- | --- | --- | --- |
| # | Cyto  band | Chr. | Gene | Expressed  allele | **Early**  combined  Z score | **Late**  combined  Z score | Result ^1^ |
| 1 | p36.32 | chr1 | TP73 | mat | 1 | -1 | -1 |
| This gene encodes a member of the p53 family of transcription factors involved in cellular responses to stress and development. Candidate gene for neuroblastoma. | | | | | | | |
| 2 | p31.3 | chr1 | DIRAS3 | pat | 1 | 1 | 0 |
| This gene is a member of the ras superfamily and it is associated with growth suppression. It appears to be a putative tumor suppressor gene whose function is abrogated in ovarian and breast cancers. | | | | | | | |
| 3 | p12 | chr2 | LRRTM1 | pat | 1 | -1 | -1 |
| May play a role during the development of specific forebrain structures by influencing neuronal differentiation and connectivity, with a possible role in intracellular trafficking within axons. It is a candidate gene for involvement in several common neurodevelopmental disorders and may have played a role in human cognitive and behavioral evolution. | | | | | | | |
| 4 | q33.3 | chr2 | ZDBF2 | pat | -1 | -1 | 0 |
| Zinc finger. | | | | | | | |
| 5 | p25.2 | chr6 | FAM50B | pat | 1 | 1 | 0 |
| Belongs to the FAM50 family. Widely expressed. Mostly abundant in testis and adult and fetal brain. | | | | | | | |
| 6 | q21 | chr6 | LIN28B | pat | -1 | -1 | 0 |
| Acts as a suppressor of microRNA biogenesis. It facilitates cellular transformation *in vitro*, and overexpression is associated with advanced disease across multiple tumor types. | | | | | | | |
| 7 | q25.3 | chr6 | SLC22A2 | mat | -1 | 1 | 1 |
| This gene is one of three similar cation transporter genes located in a cluster on chromosome 6. The encoded protein contains twelve putative transmembrane domains and is a plasma integral membrane protein. It is found primarily in the kidney, where it may mediate the first step in cation reabsorption. Polyspecific organic cation transporters in the liver, kidney, intestine, and other organs are critical for elimination of many endogenous small organic cations as well as a wide array of drugs and environmental toxins. To a lower extent, expressed in neurons of the cerebral cortex and in various subcortical nuclei. | | | | | | | |
| 8 | q32.2 | chr7 | MEST | pat | 1 | 1 | 0 |
| This gene encodes a member of the alpha/beta hydrolase superfamily. The loss of imprinting of this gene has been linked to certain types of cancer and may be due to promotor switching. The encoded protein may play a role in development. | | | | | | | |
| 9 | p12.2 | chr7 | GRB10 | isof.dep | 1 | 1 | 0 |
| This gene encodes a growth factor receptor-binding protein that interacts with insulin receptors and insulin-like growth-factor receptors. Overexpression of some isoforms of the encoded protein inhibits tyrosine kinase activity and results in growth suppression. | | | | | | | |
| 10 | q21.3 | chr7 | TFPI2 | mat | 1 | 1 | 0 |
| The protein can inhibit a variety of serine proteases including factor VIIa/tissue factor, factor Xa, plasmin, trypsin, chymotryspin and plasma kallikrein. This gene has been identified as a tumor suppressor gene in several types of cancer. | | | | | | | |
| 11 | q32.2 | chr7 | KLF14 | mat | 1 | 1 | 0 |
| This intronless gene encodes a member of the Kruppel-like family of transcription factors. The encoded protein functions as a transcriptional co-repressor, and is induced by transforming growth factor-beta (TGF-beta) to repress TGF-beta receptor II gene expression. | | | | | | | |
| 12 | p12.2 | chr7 | DDC | isof.dep. | 1 | 1 | 0 |
| The encoded protein catalyzes the decarboxylation of L-3,4-dihydroxyphenylalanine (DOPA) to dopamine, L-5-hydroxytryptophan to serotonin and L-tryptophan to tryptamine. Defects in this gene are the cause of an inborn error in neurotransmitter metabolism that leads to combined serotonin and catecholamine deficiency. | | | | | | | |
| 13 | q21.3 | chr7 | PEG10 | pat | 1 | 1 | 0 |
| This gene encodes transcripts containing two overlapping open reading frames (ORFs), RF1 and RF1/RF2, as well as retroviral-like slippage and pseudoknot elements, which can induce a -1 nucleotide frame-shift. Increased expression of this gene is associated with hepatocellular carcinomas. | | | | | | | |
| 14 | q21.3 | chr7 | PPP1R9A | mat | -1 | 1 | 1 |
| It is a regulatory subunit of protein phosphatase I and controls actin cytoskeleton reorganization. This gene is transcribed in both neuronal and multiple embryonic tissues, and it is maternally expressed mainly in embryonic skeletal muscle tissues and biallelically expressed in other embryonic tissues. | | | | | | | |
| 15 | q21.3 | chr7 | DLX5 | mat | 1 | -1 | -1 |
| This gene encodes a member of a homeobox transcription factor gene family and may play a role in bone development and fracture healing. Mutation in this gene may be associated with split-hand/split-foot malformation. | | | | | | | |
| 16 | p23.3 | chr8 | DLGAP2 | pat | 1 | 1 | 0 |
| The product of this gene is a membrane-associated guanylate kinase. The encoded protein may play a role in synapse organization and signalling in neuronal cells. This gene is biallelically expressed in the brain; however, only the paternal allele is expressed in the testis. | | | | | | | |
| 17 | p15.5 | chr11 | IGF2AS | pat | -1 | -1 | 0 |
| Insulin-like growth factor 2 antisense. Overexpressed in Wilms' tumor samples. | | | | | | | |
| 18 | p15.5 | chr11 | KCNQ1 | pat | 1 | 1 | 0 |
| This gene encodes a voltage-gated potassium channel required for repolarization phase of the cardiac action potential. Mutations in this gene are associated with hereditary long QT syndrome 1 and familial atrial fibrillation. It has been shown to be disrupted by chromosomal rearrangements in patients with BWS. | | | | | | | |
| 19 | p15.4 | chr11 | CDKN1C | mat | -1 | -1 | 0 |
| The encoded protein is a tight-binding, strong inhibitor of several G1 cyclin/Cdk complexes and a negative regulator of cell proliferation. Mutations in this gene are implicated in sporadic cancers and Beckwith-Wiedemann syndorome, suggesting that this gene is a tumor suppressor candidate. | | | | | | | |
| 20 | p15.4 | chr11 | PHLDA2 | mat | 1 | -1 | -1 |
| This gene is located in a cluster of imprinted genes on chromosome 11p15.5, which is considered to be an important tumor suppressor gene region. Alterations in this region may be associated with the Beckwith-Wiedemann syndrome, Wilms tumor, rhabdomyosarcoma, adrenocortical carcinoma, and lung, ovarian, and breast cancer. | | | | | | | |
| 21 | p13 | chr11 | WT1 | pat | 1 | -1 | -1 |
| This gene encodes a transcription factor that contains four zinc-finger motifs. It has an essential role in the normal development of the urogenital system and it is mutated in a small subset of patients with Wilm's tumors. This gene exhibits complex tissue-specific and polymorphic imprinting pattern, with biallelic, and monoallelic expression from the maternal and paternal alleles in different tissues. | | | | | | | |
| 22 | q22.3 | chr11 | ZC3H12C | pat | 1 | -1 | -1 |
| Zinc finger. | | | | | | | |
| 23 | q14.2 | chr13 | RB1 | mat | 1 | 1 | 0 |
| The protein encoded by this gene is a negative regulator of the cell cycle and was the first tumor suppressor gene found. The encoded protein also stabilizes constitutive heterochromatin to maintain the overall chromatin structure. Defects in this gene are a cause of childhood cancer retinoblastoma (RB), bladder cancer, and osteogenic sarcoma. | | | | | | | |
| 24 | q32.2 | chr14 | DLK1 | pat | 1 | -1 | -1 |
| This gene encodes a transmembrane protein containing six epidermal growth factor repeats. The protein is involved in the differentiation of several cell types, it is also thought to be a tumor suppressor. It is one of several imprinted genes located in a region of on chr 14q32. Certain mutations in this imprinted region can cause phenotypes similar to maternal and paternal uniparental disomy of chromosome 14 (UPD14). | | | | | | | |
| 25 | q11.2 | chr15 | SNRPN | pat | -1 | 1 | 1 |
| The protein plays a role in pre-mRNA processing, possibly tissue-specific alternative splicing events. Alternative splicing or deletion caused by a translocation event in this paternally-expressed region is responsible for Angelman syndrome or Prader-Willi syndrome due to parental imprint switch failure. | | | | | | | |
| 26 | q11.2 | chr15 | UBE3A | mat | 1 | 1 | 0 |
| This gene encodes an E3 ubiquitin-protein ligase, part of the ubiquitin protein degradation system. This imprinted gene is maternally expressed in brain and biallelically expressed in other tissues. Maternally inherited deletion of this gene causes Angelman Syndrome, characterized by severe motor and intellectual retardation, ataxia, hypotonia, epilepsy, absence of speech, and characteristic facies. The protein also interacts with the E6 protein of human papillomavirus types 16 and 18, resulting in ubiquitination and proteolysis of tumor protein p53. | | | | | | | |
| 27 | q12 | chr15 | ATP10A | mat | 1 | -1 | -1 |
| The protein encoded by this gene belongs to the family of P-type cation transport ATPases, and to the subfamily of aminophospholipid-transporting ATPases. It maps within the most common interval of deletion responsible for Angelman syndrome. | | | | | | | |
| 28 | q11.2 | chr15 | NDN | pat | 1 | 1 | 0 |
| This intronless gene is located in the Prader-Willi syndrome deletion region. It is an imprinted gene and is expressed exclusively from the paternal allele. Studies in mouse suggest that the protein encoded by this gene may suppress growth in postmitotic neurons. | | | | | | | |
| 29 | p13.3 | chr16 | ZNF597 | mat | 1 | -1 | -1 |
| Zinc finger. | | | | | | | |
| 30 | q21.1 | chr18 | TCEB3B | mat | 1 | -1 | -1 |
| This gene encodes the transcriptionally active subunit of the SIII (or elongin) transcription elongation factor complex. This complex acts to increase the rate of RNA chain elongation by RNA polymerase II by suppressing transient pausing of the polymerase at many sites along the DNA template. Whereas a related protein with similar function, elongin A, is ubiquitously expressed, the encoded protein is specifically expressed in the testis, suggesting it may have a role in spermatogenesis. | | | | | | | |
| 31 | q13.43 | chr19 | ZIM2 | pat | 1 | 1 | 0 |
| Zinc finger. | | | | | | | |
| 32 | q13.42 | chr19 | NLRP2 | mat | 1 | -1 | -1 |
| NALP proteins are involved in the activation of caspase-1 by Toll-like receptors. They may also be involved in protein complexes that activate proinflammatory caspases. | | | | | | | |
| 33 | q11.23 | chr20 | BLCAP | isof. dep | -1 | -1 | 0 |
| This gene encodes a tumor suppressor protein that reduces cell growth by stimulating apoptosis. This gene is imprinted in brain where it is differentially expressed from different promoters. Transcription from the upstream promoter occurs preferentially on the maternal allele, and transcripts are preferentially expressed from the downstream promoter on the paternal allele. | | | | | | | |
| 34 | q13.32 | chr20 | GNAS | isof. dep | 1 | 1 | 0 |
| This locus has a highly complex imprinted expression pattern. It gives rise to maternally, paternally, and biallelically expressed transcripts that are derived from four alternative promoters and 5' exons. Some transcripts contain a differentially methylated region (DMR) at their 5' exons, and this DMR is commonly found in imprinted genes and correlates with transcript expression. One of the transcripts produced from this locus, and the antisense transcript, are paternally expressed noncoding RNAs, and may regulate imprinting in this region. | | | | | | | |
| 35 | q13.32 | chr20 | hsa-mir-296:214 (MIR*296)* | pat | 1 | 1 | 0 |
| miR-296 has been named an "angiomiR" due to being characterised as a microRNA which regulates angiogenesis and is therefore thought to have a specific role in carcinogenesis. It achieves this by targeting HGS mRNA, reducing its expression in endothelial cells which then results in greater number of VEGF receptors. miR-296 has predicted target sites in the transcription factor NANOG and may also contribute to carcinogenesis by dysregulating p53. | | | | | | | |
| 36 | q11.21 | chr22 | DGCR6L | Unknown | -1 | -1 | 0 |
| This gene, the result of a duplication at this locus, is one of two functional genes encoding nearly identical proteins that have similar expression patterns. The product of this gene is a protein that shares homology with the human laminin gamma-1 chain that functions in cell attachment and migration. This gene is located in a region of chromosome 22 implicated in the DiGeorge syndrome. | | | | | | | |
| 37 | q11.21 | chr22 | DGCR6 | unknown | -1 | -1 | 0 |
| This gene is a candidate for involvement in DiGeorge syndrome pathology and in schizophrenia. | | | | | | | |

^1^ Methylation pattern: 0: Unvaried; -1: Non-methylated in late compared to early; +1: Methylated in late compared to early
